# Discovery of Golgi membrane-associated degradation (GOMED) pathway: a focus on 15 years of ultrastructural analyses

**DOI:** 10.1093/jmicro/dfaf023

**Published:** 2025-05-13

**Authors:** Satoko Arakawa, Hirofumi Yamaguchi, Shigeomi Shimizu

**Affiliations:** Ochanomizu Research Facility (ORF), Bioscience Center, Research Infrastructure Management Center, Institute of Science Tokyo, 1-5-45 Yushima, Bunkyo-ku, Tokyo 113-8510, Japan; Department of Pathological Cell Biology, Advanced Research Initiative, Institute of Science Tokyo, 2-3-10 Kandasurugadai, Chiyoda-ku, Tokyo 101-0062, Japan; Department of Pathological Cell Biology, Advanced Research Initiative, Institute of Science Tokyo, 2-3-10 Kandasurugadai, Chiyoda-ku, Tokyo 101-0062, Japan

**Keywords:** Golgi, autophagy, GOMED, mitophagy, Wipi3, neurodegeneration

## Abstract

In this review, we focus on the ultrastructural characteristics of the Golgi membrane-associated degradation (GOMED) pathway, which have been clarified by electron microscopy, and highlight recent advances in the elucidation of its molecular mechanism and physiological roles. The discovery of GOMED, an Atg5/Atg7-independent degradation pathway that differs from canonical autophagy in membrane origin, stimuli and substrate specificity, has substantially expanded our understanding of intracellular degradation systems. In 2009, we identified GOMED as a novel, evolutionarily conserved autophagic pathway and demonstrated its role in intracellular degradation across eukaryotes, from yeast to mammals. We identified the conserved protein Hsv2/Wipi3 as an essential GOMED protein, which translocates to the *trans*-Golgi upon induction and remodels Golgi membranes into cup-shaped structures that engulf cytoplasmic components for lysosomal degradation. These processes contribute to organelle and secretory granule turnover, as well as mitochondrial clearance during erythroid differentiation. Moreover, neuronal-specific ablation of Wipi3 in mice causes severe cerebellar degeneration, implicating GOMED in tissue development and homeostasis. As these mechanisms are associated with diseases, such as neurodegenerative disorders and cancer, GOMED mechanisms should also be considered when establishing therapeutic strategies for these diseases.

## Graphical Abstract

**Figure:**
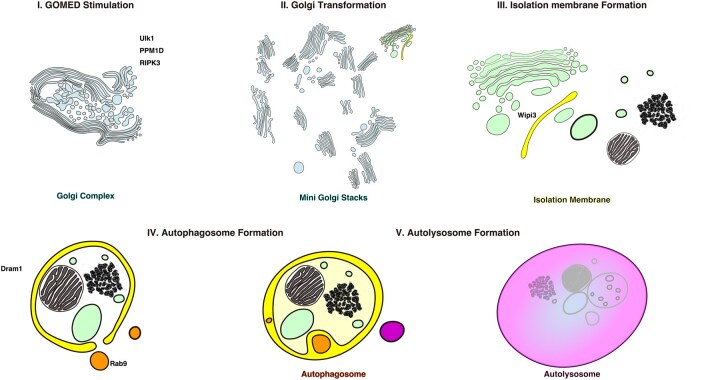
Morphological and Mechanistic Insights into GOMED: A Golgi-Membrane Degradation Pathway I. GOMED Stimulation Golgi membrane-associated degradation (GOMED) is induced by various cellular stresses, such as DNA damage and the inhibition of cargo transport within the Golgi owing to an overloaded transport pathway. Under such stressed conditions, GOMED degrades proteins that accumulate and stagnate in the Golgi. Chemical agents such as the DNA-damaging drug etoposide and 1,3-cyclohexanebis - a compound that interferes with coatomer binding to Golgi membranes, thereby inhibiting anterograde trafficking - can also induce GOMED[11,12]. Key regulatory steps involve Ulk1 phosphorylation dynamics; see details in refs. 23, 50, and 51. II. Golgi Transformation Upon GOMED induction, the Golgi undergoes morphological reorganization. In animal cells, the normally contiguous Golgi stacks fragment into dispersed ministacks, concomitant with reduced phosphatidylinositol 4-phosphate [PI(4)P] levels on Golgi membranes[12]. III. Isolation Membrane Formation The trans-Golgi of the ministack Golgi separates from the stack structure, forming an isolation membrane. At this stage, dephosphorylated Wipi3 translocates from the cytoplasm to the Golgi[42]. The isolation membrane subsequently engulfs mitochondria, secretory granules, and other substrates. However, the tethering molecules and adaptors involved in this process remain unidentified. IV. Autophagosome Formation The isolation membrane encloses the substrates, forming a double-membrane autophagosome. This process requires Dram and Rab9[11,49]. V. Autolysosome Formation The double-membraned autophagosome fuses with lysosomes to form single-membraned autolysosomes, where the enclosed contents are degraded. Further fusion between autolysosomes, as well as between autolysosomes and endosomes, contribute to autolysosome enlargement.

## Teaser

This review highlights the ultrastructural characteristics and molecular mechanism of GOMED, an evolutionarily conserved autophagic pathway. Via reorganizing Golgi membranes, GOMED eliminates mitochondria during erythroid differentiation, and maintains neuronal homeostasis. Golgi-translocating protein Wipi3 is essential for GOMED, with its dysfunction linked to cerebellar degeneration, emphasizing GOMED’s role in physiological functions.

## Introduction

Autophagy is a cellular process that degrades various intracellular components, including proteins and organelles [1–3]. In 1962, Ashford and Porter pioneered electron microscopy (EM) studies that characterized the morphology of canonical autophagy for the first time, revealing double-membrane-bound autophagosomes that mature into single-membrane autolysosomes [4,5]. Their initial observations were made in the rat liver during perfusion with glucagon, a peptide hormone secreted during hypoglycemia. During canonical autophagy, double membrane structures called the isolation membranes expand to engulf organelles, such as mitochondria and peroxisomes, forming autophagosomes. The autophagosomes subsequently fuse with lysosomes, becoming single-membrane autolysosomes that degrade subcellular components using lysosomal enzymes [1,2].

Pioneering genetic screens in yeast identified the genes encoding components of the molecular machinery of autophagy, called autophagy-related (ATG) genes, independently by the Ohsumi group, Klionsky group and Thumm group [6–9]. Almost half of the Atg proteins play crucial roles in the formation of sequestration membranes and autophagosomes. Among these, Atg5 and Atg7 have been extensively studied using knockout models, providing important insights into their roles in autophagy. Canonical autophagy has now been shown to utilize the endoplasmic reticulum (ER) membrane as a crucial source of the isolation membrane, to which Atg9 vesicles are partially added in their autophagosomes [10].

**Fig. 1.**
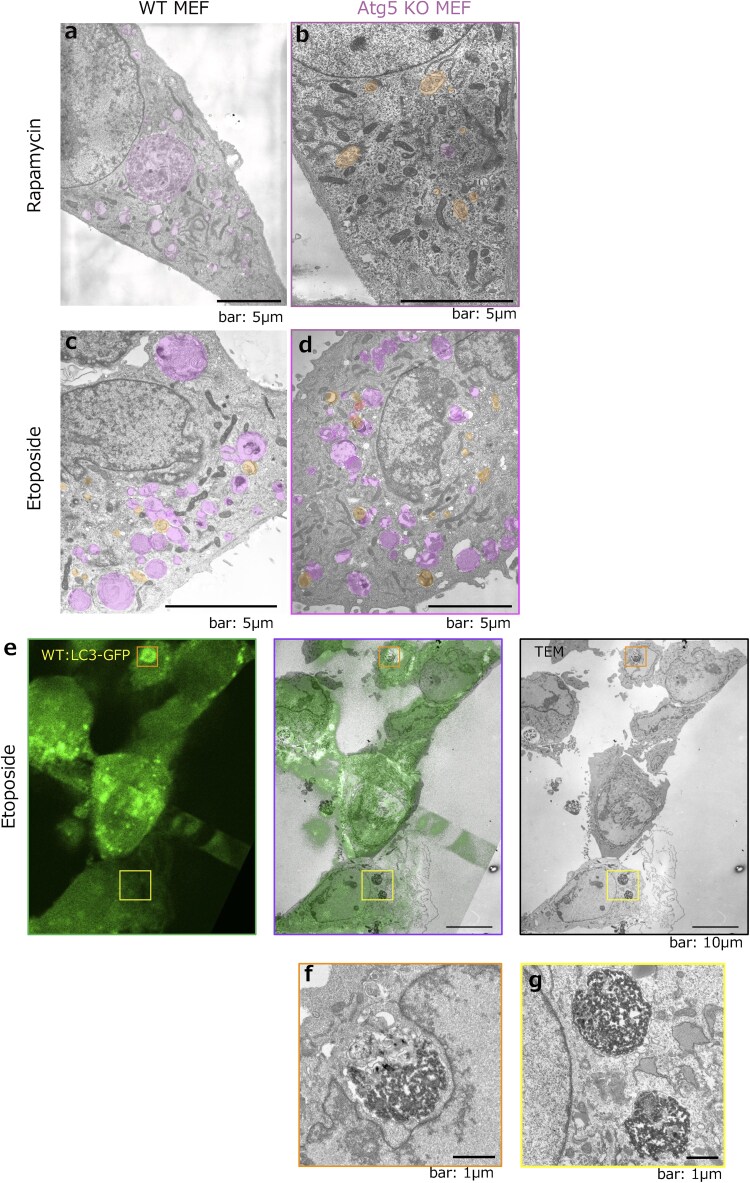
Discovery of GOMED, an Atg5/Atg7-independent alternative autophagy process. EM of WT (a) and *Atg5^KO^* MEFs (b) treated with 1 µM rapamycin or 10 µM etoposide (c, d) for 18 h. Scale bar = 5 μm. Areas highlighted in magenta represent autolysosomes, and those highlighted in orange indicate autophagosomes. Both canonical and alternative autophagy (GOMED) were induced in etoposide-treated (e–g) MEFs. WT MEFs were transfected with GFP-LC3, cultured on coverslips, treated with 10 µM etoposide for 18 h, and then fixed with glutaraldehyde and paraformaldehyde. Following observation of GFP-LC3 fluorescence, the same cells were fixed with OsO_4_ and observed by EM. The scale bar in the magnified photo is 1 µm. Both GFP-LC3-positive (canonical) autophagy (f) and GFP-LC3-negative (alternative) autophagy, GOMED (g), were activated in etoposide-treated WT MEFs. (a), (b), and (e)–(g) were modified from Supplementary Fig. 2b and Fig. 11 in ref. [11], with permission from *Nature*. (c) and (d) were obtained from the same experiments as those shown in Supplementary Fig. 2c of ref. [11].

However, when we observed mammalian cells that lack Atg5 or Atg7 by EM, we found that these cells still contained autophagosomes and autolysosomes even in the absence of canonical autophagy (Fig. 1). We named this alternative autophagy GOMED (Golgi membrane-associated degradation), during which the Golgi apparatus supplies membranes for autophagic sequestration rather than the ER [9,10]. During GOMED, the membrane from Golgi stacks remodels into an isolated membrane enclosing the material destined for lysosomal degradation [11,12].

The discovery of GOMED has substantially expanded our understanding of intracellular degradation systems. Our research group was the first to identify GOMED through ultrastructural analyses using EM. This finding underscores the limitations of relying solely on canonical autophagy markers, such as LC3 (microtubule-associated protein 1 light chain 3) [13] and p62(SQSTM1/Sequestosome-1) accumulation [14], to evaluate autophagy activity. GOMED and other forms of alternative autophagy differ from canonical autophagy in several key aspects, including the origin of the membranes used, the stimuli that induce the pathway and the specific substrates targeted for degradation.

Furthermore, the discovery of GOMED has important implications for understanding and treating diseases, such as neurodegenerative disorders [15,16], cancer [17,18] and cardiovascular diseases [19–21]. The existence of GOMED suggests that targeting canonical autophagy alone may be insufficient for therapeutic purposes, as compensatory mechanisms, including GOMED, may be activated. Despite these advances, many aspects of the molecular mechanisms of GOMED remain unclear, including its crosstalk with canonical autophagy and its role in disease pathogenesis.

In this review, we summarize recent advances in our elucidation of the molecular mechanisms and physiological roles of GOMED, with a particular focus on its ultrastructural characteristics as revealed by EM. For detailed information on the significance of GOMED/alternative autophagy within the broader context of the progression of autophagy research, we recommend consulting other reviews [15,22–25]. The discovery of GOMED has substantially advanced our understanding of the complexity and adaptability of cellular stress responses, suggesting that cells have evolved diverse mechanisms to maintain homeostasis and adapt to various environmental challenges.

As the field of GOMED/alternative autophagy continues to evolve, we believe that understanding the morphological characteristics of these pathways will be crucial for elucidating their molecular mechanisms. By providing a detailed account of its unique features and physiological roles, we aim to contribute to the broader understanding of GOMED and its importance in autophagy research.

## Discovery of Atg5/Atg7-independent alternative autophagy

Mice lacking Atg5 or Atg7 die within a day of birth [26,27]. However, cultured cells lacking Atg5 or Atg7 grow without any problems, and embryos lacking either Atg5 or Atg7 have no abnormalities. Because autophagy is essential for maintaining homeostasis, we hypothesized that specific pathways might compensate for canonical autophagy.

Canonical autophagy can be induced by starvation or by treatment with rapamycin (Fig. 1a), but it is known that autophagy cannot be induced in cells lacking Atg5 or Atg7 (Fig. 1b). However, using EM, we found that addition of the anticancer drug etoposide, but not rapamycin, resulted in the appearance of autophagosomes and autolysosomes in both wild type (WT) and Atg5-deficient (*Atg5^KO^*) mouse fibroblast (MEF) cells (Fig. 1c and d). Electron and light microscopy analyses of LC3 (Fig. 1e), a marker of canonical autophagy, showed both LC3-positive (Fig. 1f) and LC3-negative (Fig. 1g) vacuoles in WT MEFs following etoposide treatment. The size and number of autolysosomes were the same in both cell types [11]. This suggests that mammalian cells have an Atg5-independent type of macroautophagy. Some other groups have also reported an Atg5-independent type of macroautophagy [16,19,28–31].

## The isolation membrane is derived from the *trans*-Golgi membrane during autophagy induction in *Atg5^KO^* MEFs

**Fig. 2.**
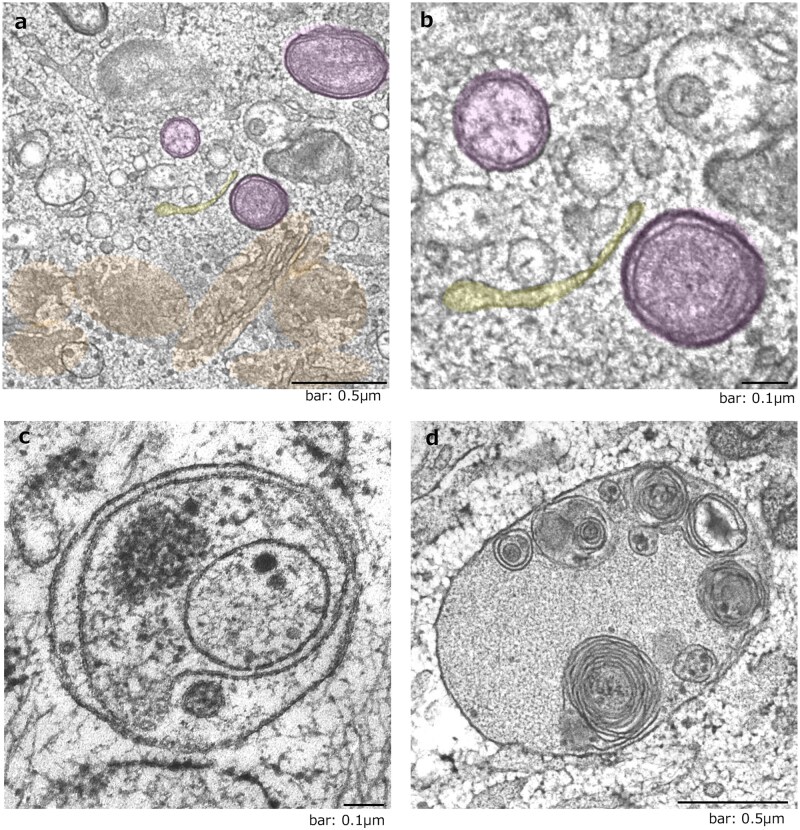
The isolation membrane originates from the *trans*-Golgi membrane during GOMED induction. *Atg5^KO^* MEFs were treated with 10 µM etoposide for 18 h, and fixed using quick freezing and freeze-substitution methods (ref. [11]). (a) and (b) The isolation membrane (yellow cisternae) was derived from the Golgi ministacks. Autophagosomes are shown in magenta. (c) Autophagosome. (d) Autolysosome. (a)–(d) were obtained from the same experiments of Fig. 2a of ref. [12].

We prepared samples from earlier time points, during 6–12 h after treatment of etoposide, to capture autophagosome formation. We then observed that the isolation membrane derived from the Golgi membrane from the ministack Golgi (Fig. 2a and b). Other autophagic structures, such as autophagosomes (Fig. 2a–c) and autolysosomes (Fig. 2d), were also found around the Golgi stacks. We then identified an alternative autophagy mechanism that is independent of Atg5 and Atg7. It is distinct from canonical autophagy in the source of the isolation membrane. We named this alternative autophagy GOMED.

## GOMED is conserved from yeast to mammalian cells

Following the discovery of GOMED in mammalian cells, the next question was whether GOMED is conserved among eukaryotes. We predicted that GOMED would not be found in the yeast *Saccharomyces cerevisiae*, as their Golgi cisternae are typically dispersed in the cytoplasm, and it is difficult to observe a stack-style pattern [32–37].

**Fig. 3.**
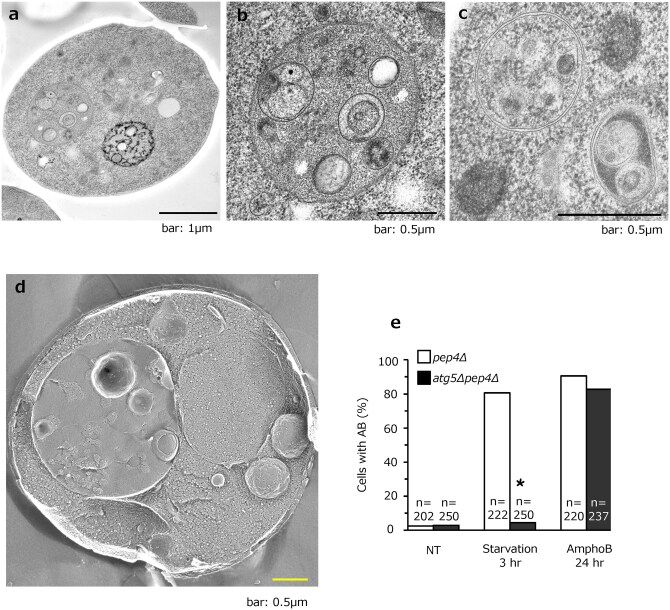
Induction of AB structures in *atg5Δpep4Δ* cells treated with AmphoB. Representative images of thin sections of frozen-substituted material (a–c) and quick-frozen replicas (d) are shown. ABs accumulated in the vacuole (a, b, d). ABs containing lipid particles and mitochondria were observed in vacuole cross-sections (b). An autophagosome (AP) with a double-membrane compartment containing a ribosome and Golgi granule was present in the cytosol (c). (e) Cells containing AB structures were counted under EM (no treatment [NT]: *n* = 20, AmphoB (2.5 µg/ml, 24 h): *n* = 192). *P < 0.01 versus NT. (a) and (b) are modified from Fig. 1J of ref. [12], and (c) was obtained from the same experiment of (a) and (b). (d) and (e) were modified from Fig 1I and H, respectively of ref. [12], with permission from *EMBO J*.

To test whether Atg5-independent autophagy exists in yeast, we first added various chemicals to Atg5-deficient yeast cells. We tested many chemicals, but it was difficult to induce autophagy without the *Atg5* gene. We then considered the fact that GOMED can be induced by adding anticancer drugs to *Atg5^KO^* MEFs at concentrations that do not induce cell death. Therefore, we decided to try adding antifungal drugs to yeast at concentrations that would not induce cell death [12,38], and found that *atg5-*deficient cells can induce GOMED in the presence of the antifungal drug amphotericin B1 (AmphoB) (Fig. 3a–d); double-membrane autophagosomes are formed in the cytoplasm (Fig 3c), and these dock and fuse with vacuoles, enabling their contents to enter the vacuoles [12]. Addition of AmphoB to WT cells also induced autophagy, and the rate of autophagy induction was confirmed to be similar between WT and *atg5*-deficient cells, by counting the proportion of cells with autophagic bodies (ABs) within their vacuoles (Fig. 3e).

## Yeast has stacked Golgi cisternae during the induction of GOMED

**Fig. 4.**
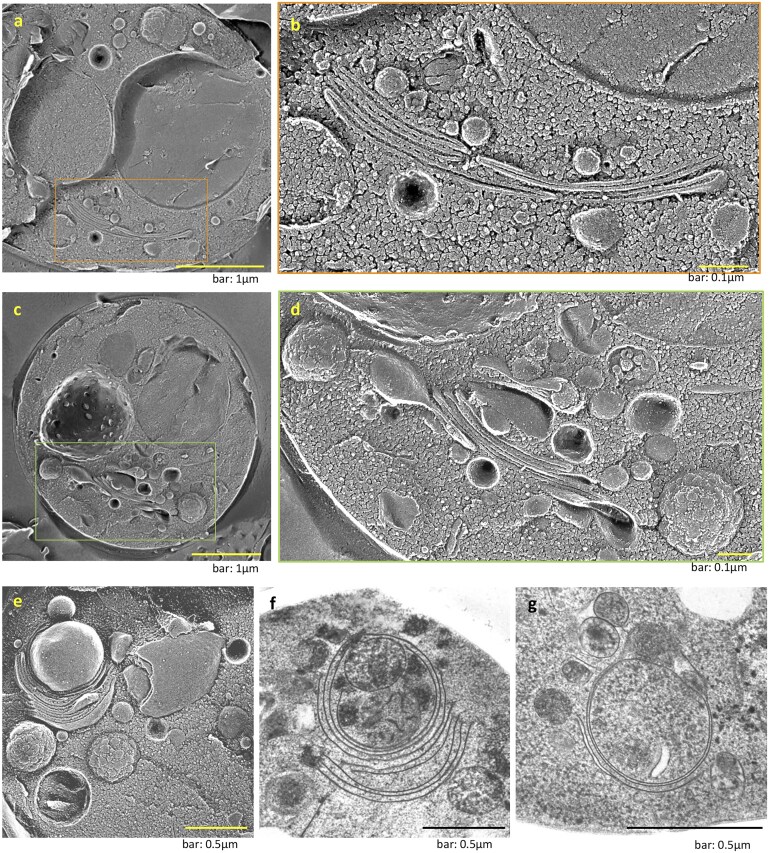
Formation of Golgi stacks induced by AmphoB and their involvement in GOMED. (a–d) Stacked Golgi cisternae are observed in quick-frozen replicas (a–d) of *atg5Δpep4Δ* cells treated with AmphoB (2.5 mg/ml). (b, d) The magnified images depict the area indicated by the square in (a) and (c). Scale bars = 1 µm (a, c) and 0.1 µm (b, d). (e–g) Formation of autophagic double-membrane compartments from the stacked Golgi. A quick-frozen replica (e) and a substituted thin section (f, g) are presented. Scale bars = 0.5 µm. The distal Golgi stacks are extended, curved and form autophagosome structures (e, f). Mitochondria are encircled by the Golgi membrane (f). (g) The autophagosome structure was separated from the Golgi stacks. (a) and (b) were modified from Fig. 2C; (c) and (d) were modified from Fig. 2A and 2B, respectively; (e) and (f) were modified from Fig. 2E and 2F, respectively of ref. [12] with permission from *EMBO J*. (g) was obtained from the same experiments as those shown in Fig. (f).

Temporal EM profiling revealed the striking remodeling of Golgi in yeast after GOMED induction with AmphoB. After 9 h of AmphoB treatment, stacked Golgi structures were observed (Fig. 4a–d), although yeast Golgi are typically dispersed under normal conditions [32–37]. From *cis*-to-*trans*, each Golgi stack contains a number of structures that appear to have protein granules that enlarge toward the outermost cisternae (Fig. 4d). Moreover, there is an incremental accumulation and growth in size of the protein granules at the edge of the cisternae. We also found that the outermost layer of the Golgi stack, or the second membrane from the outer layer, curves substantially over time to become an autophagosome that engulfs organelles, such as protein granules and mitochondria. In contrast, no *trans*-Golgi networks were found outside the Golgi stacks (Fig. 4e–g).

## Reduction of Golgi phosphatidylinositol-4-phosphate level induces the inhibition of secretion and promotes GOMED

AmphoB is known to directly interfere with ergosterol [38], and the amount of ergosterol in the ER regulates phosphatidylinositol-4-phosphate [PI(4)P] levels at the Golgi membrane [39]. We then analyzed Golgi PI(4)P reduction at the Golgi membrane following AmphoB treatment [12].

*pik1-83* is a temperature-sensitive yeast mutant that loses its Golgi PI(4)P kinase activity at or above 37°C [40]. Localization analysis confirmed the loss of PI(4)P at the Golgi in *pik1-83/atg5Δ* cells at 37°C. In these cells, the accumulation of O-glycosylated Hsp150 indicates a failure of trafficking from the Golgi to the plasma membrane (PM) [12].

**Fig. 5.**
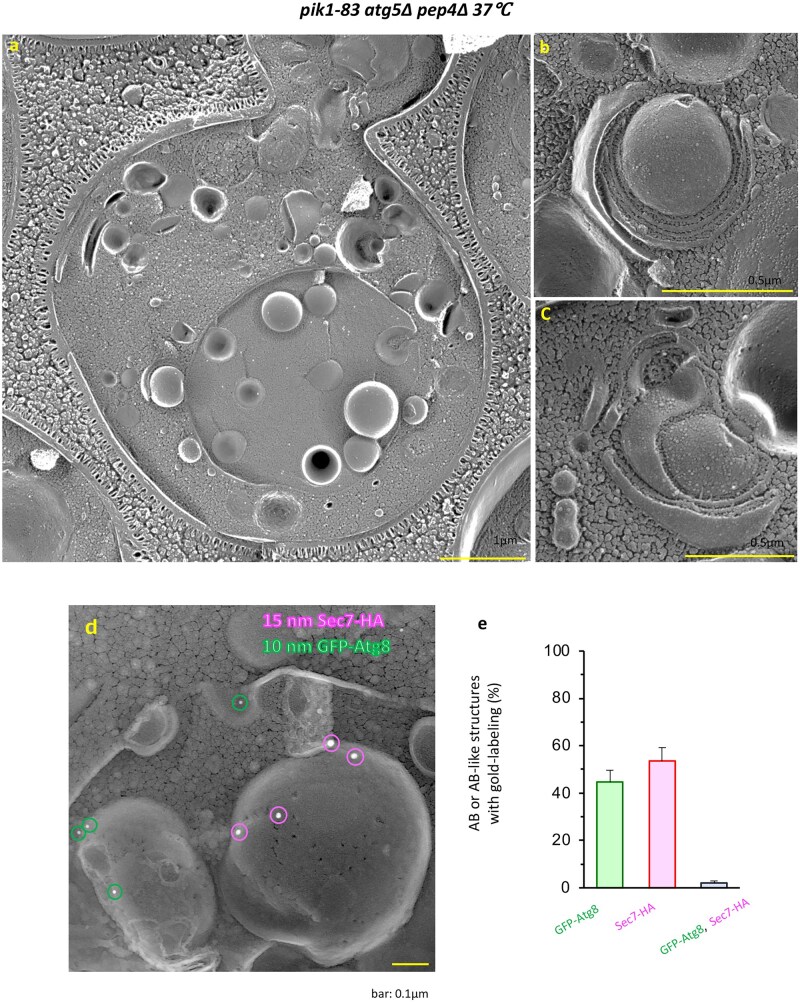
Induction of GOMED by the deletion of Golgi PI(4) kinase. (a) Representative EM image of *pik1-83/atg5Δpep4Δ* cells at 3 h after a temperature shift from 25°C to 37°C, followed by freeze-replica method. AB-like structures were observed in the vacuole. Scale bar = 1 µm. (b, c) After the temperature shift, cells demonstrated stacked Golgi and autophagosomes formed from the *trans*-Golgi, similar to AmphoB-treated *atg5Δpep4Δ* cells. (d) ABs consist of two types in Atg5-positive GOMED-induced cells. GFP-Atg8/Sec7-HA-expressing *pik1-83* cells were incubated at 37°C for 3 h, followed by freeze-replica immunolabeling for HA (15-nm gold particles) and GFP (10-nm gold particles). Two types of structures were identified in the vacuoles, namely, GFP-Atg8-positive ABs and Sec7-HA-positive AB-like structures, indicating the induction of GOMED in WT yeast cells. Scale bar = 0.1 µm. (e) Percentages of GFP-Atg8-positive ABs and Sec7-HA-positive AB-like structures were calculated (mean ± SEM, *n* = 6 experiments, 1081 structures). Few structures contained both signals. (a)–(c) were obtained from the freeze-replica methods [41] as those shown in Fig. 4. (d) and (e) are modified versions of Fig. EV3B and EV3C of ref. [12], with permission from *EMBO J*.

We analyzed these *pik1-83/atg5Δpep4Δ* cells by platinum replica made from quick-freezing and fracturing method [41]. ABs were found in the vacuoles induced after temperature changes, as observed by EM (Fig. 5a–c). In the cytoplasm, we also observed Golgi stacks and autophagosome formation from the stacked Golgi cisternae (Fig. 5b and c).

## AB originates from two types of membranes in WT yeast

Immunogold labeling of the yeast replica (immuno-replica) confirmed the existence of Sec7-positive (Golgi marker) and Atg8-positive (LC3 homolog) ABs, whereas double-positive structures were never observed. Analysis using various markers suggests that yeast also has two types of ABs; namely, Atg8-positive structures produced by canonical autophagy, and Golgi-derived Sec7-positive structures produced by GOMED (Fig. 5d and e).

## Identification of genes essential for GOMED using yeast genetics

**Fig. 6.**
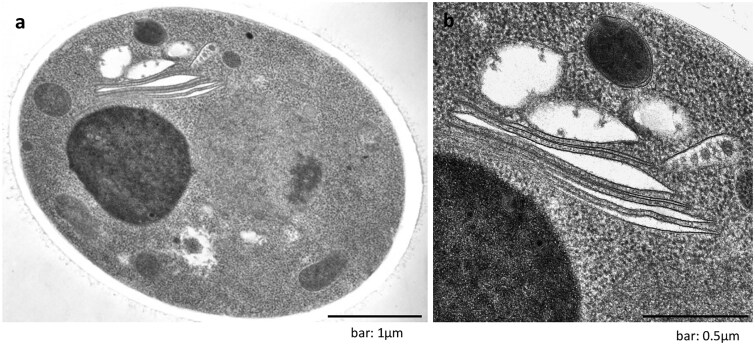
Requirement of Hsv2 for the AmphoB-induced GOMED response. (a, b) *hsv2Δatg5Δpep4Δ* yeast cells were treated with AmphoB (2.5 μg/ml for 24 h) and observed by EM. In *hsv2Δatg5Δpep4Δ* cells, stacked and swollen Golgi membranes were observed. Autophagosome and AB structures were not observed in the cytosol and vacuoles (see ref. [42], Fig. 1g and h). Scale bars = 1 µm (a); 0.5 μm (b). (a) and (b) were obtained from the same experiments as those shown in Fig. 1f and g of ref. [42] but were images of different cells.

We have shown that the GOMED system requires Ulk1, Dram and Rab9 in mammals [11]. To further identify molecules involved in GOMED, we investigated a database of synthetic lethal genes for canonical autophagy-associated genes in yeast, as the simultaneous loss of molecules involved in both types of autophagy is expected to reduce cell viability [12,42]. To screen for GOMED progression in yeast cells, we treated yeast cells with the antifungal drug AmphoB. This screening approach successfully led to the identification of *HSV2* as an essential gene in the GOMED pathway [42]. In *hsv2*-deficient cells, Golgi bodies were found to be stacked, but their lumina were swollen, without any changes in the curvature of their *trans*-Golgi membranes (Fig. 6a and b). This result suggests that Hsv2 may play a role in inducing the curvature necessary for the *trans*-Golgi membrane to become autophagosomes.

## *HSV2* ortholog *Wipi3* is an essential mammalian GOMED gene

**Fig. 7.**
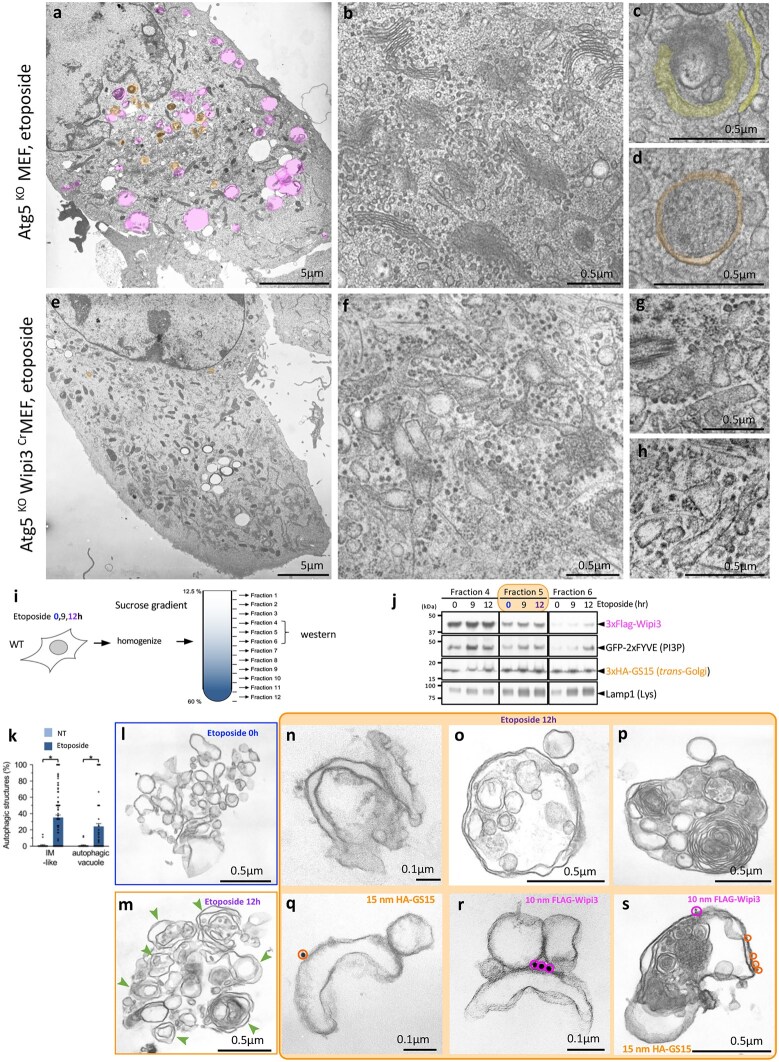
Wipi3 is essential for etoposide-induced GOMED. (a, e) Electron micrographs of the indicated MEFs treated with etoposide (10 μM) for 18 h. In *Atg5^KO^* MEFs, numerous autolysosomes were present in the cytosol, but not in *Atg5^KO^ Wipi3^Cr^* MEFs. Scale bars = 5 µm. The areas shown in magenta represent autolysosomes, and those in orange indicate autophagosomes. (b–d) After etoposide treatment (10 μM for 12 h), Golgi ministacks appeared in the cytosol of control cells and contributed to the formation of autophagosome (*Atg5^KO^* MEFs), but the stacked structures were disorganized, and rod-shaped structures were present in the cisternae of *Atg5^KO^ Wipi3^Cr^* MEFs (f–h). Samples were prepared using the quick freeze-substitution method. Scale bars: 5 µm (a, e); 0.5 µm (b–d, f–h). (i–s) Localization of Wipi3 and GS15 on isolated autophagic GOMED membranes, indicating the successful fractionation of *trans*-Golgi membranes. (i) *Atg5^KO^* MEFs were treated with etoposide (10 μM), lysed in isotonic buffer and fractionated by sucrose-gradient centrifugation. The expression of each molecule was analyzed by western blotting (j). (k) The populations of isolation membrane (IM)-like and autophagic structures per total structures were calculated. Data are shown as the mean ± SEM (NT: *n* = 25 structures; Etoposide: *n* = 122 structures). Representative electron micrographs of fraction 5 from untreated (l) and etoposide-treated *Atg5^KO^* MEFs (m). Arrowheads indicate autophagosome-like structures. Representative IM-like (n, q, r) and autophagosome-like (o) and autolysosome-like (p, s) structures are shown in the right panels. (q–s) Immunoelectron micrographs of fraction 5 from etoposide-treated *Atg5^KO^* MEFs. HA-GS15 and Flag-Wipi3 were recognized by 15-nm gold-conjugated anti-HA antibodies and 10-nm gold-conjugated anti-Flag antibodies, respectively. In (k), *P < 0.01 vs the value of untreated MEFs (IM-like: *P* < 0.0001, autophagic vacuole (autophagosome-like and autolysosome-like structures): *P* = 0.0083). (a) and (e) are obtained from the same experiments but were images of different cells of Fig. 2a and b of ref. [42], and (b) and (f) are modified versions of Fig. 3a and b from ref. [42], respectively. (c) and (d) were obtained from the same experiments as those shown in (b). (j) and (k) are modified versions of Fig. 4a and d, respectively from ref. [42]. (l), (m), (n), (o) and (p) are modified versions of Fig. 4b and c from ref. [42]. (q), (r) and (s) are modified versions of Fig. 4e from ref. [42].

The mammalian ortholog of *HSV2* is a gene called *Wipi3* [42,43]. We then investigated the morphological changes in MEF cells lacking Wipi3 [42]. When control cells were treated with the DNA-damaging agent etoposide to induce GOMED, many autophagic structures were observed in the cytoplasm (Fig. 7a). Simultaneously, the Golgi was transformed into a ministack (Fig. 7b–d), and *trans*-Golgi-derived isolation membranes were formed (Fig. 7b), followed by autophagosome formation (Fig. 7d). In contrast, autophagic structures were barely visible in cells lacking Wipi3 (Fig. 7e). In the perinuclear region, we found that the Golgi of these cells undergoes segmentation into several Golgi ministacks. However, in contrast to control cells, the cisternae became swollen and disrupted the stacked structure (Fig. 7f–h). Notably, these swollen cisternae are unable to transition into isolation membranes. This observation provides compelling evidence that Wipi3 plays a role in promoting the Golgi membrane to form the isolation membrane [42].

## Wipi3 localizes to the *trans*-Golgi and autophagosomal membranes after GOMED induction

We next investigated the localization of Wipi3 following the induction of GOMED [42]. Cells were homogenized using a loose-fitting Dounce homogenizer in isotonic buffer followed by sucrose gradient centrifugation (Fig. 7i). The *trans*-Golgi and lysosomes were recovered in fractions 4–6 using the *trans*-Golgi marker GS15, and the lysosomal marker Lamp1 (Fig. 7j). Wipi3 levels in these fractions increased with time after etoposide treatment. We then analyzed the fraction 5 by EM (Fig 7k–s). In nontreated cells, the fraction primarily consisted of small, round, single-membrane vesicles (Fig. 7l). However, after etoposide treatment, autophagic structures, including cup-shaped structures (Fig 6n), double-membrane autophagosomal structures (Fig. 7o) and autolysosomal vacuoles containing multilamellar bodies (Fig. 7p) were predominant in this fraction 5 (Fig. 7k and m). Importantly, immuno-EM analysis revealed the presence of Wipi3 and GS15 antibodies on the surface of the isolation membrane-like structures (Fig. 7q and r) and autolysosomes (Fig. 7s). In conclusion, we confirmed the localization of Wipi3 on Golgi-derived isolation membranes and the subsequent formation of autophagic structures. Following GOMED induction, cytoplasmic Wipi3 localizes to the *trans*-Golgi membrane and autophagic structures [42].

## Physiological function of GOMED in erythrocyte maturation during the embryonic stage

Next, we will present insights into the physiological functions of GOMED, as demonstrated by the analysis of *Atg5^KO^* mice and *Wipi3^KO^* mice [11,12,44]. We first focused on *Atg5^KO^* mice[11]. These mice die within a day after birth [27], but they grow similarly to WT mice during the embryonic period and show little difference in their morphology. Based on this observation, we hypothesized that alternative autophagy, i.e. GOMED, might be induced in *Atg5^KO^* mice instead of canonical autophagy. We therefore carried out a thorough investigation of various organs of these mice and found that GOMED was most strongly induced in the fetal liver [11,44].

**Fig. 8.**
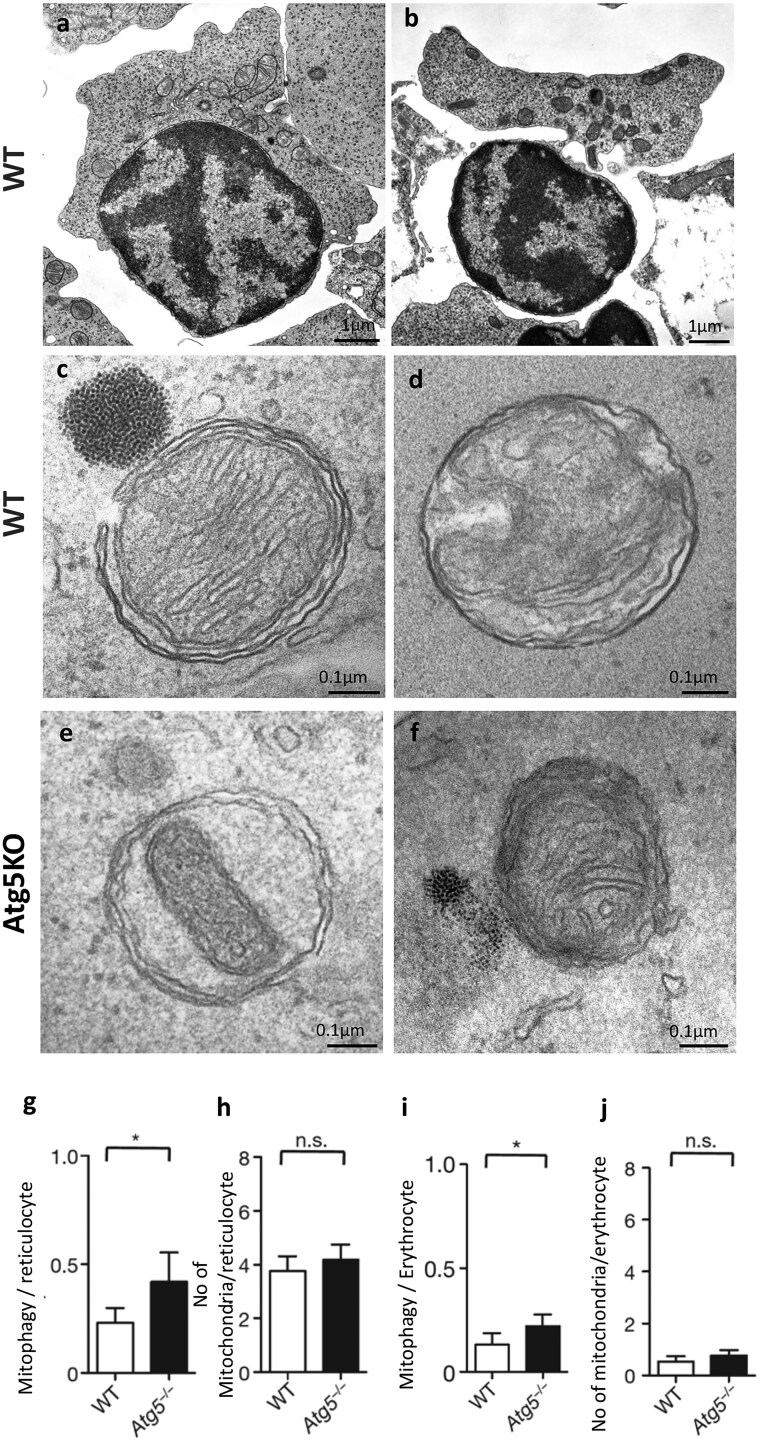
Physiological roles of GOMED during erythrocyte maturation in the fetal liver of mice. (a, b) Erythrocytes undergo organelle clearance during terminal differentiation after enucleation. Ultrastructural analysis of the WT mouse fetal liver on embryonic day (E)14.5, when hematopoiesis occurs, showed that autophagic structures (autophagosomes and autolysosomes) in reticulocytes engulf and digest mitochondria. (c, e) Autophagosomes with double membranes encircling mitochondria in reticulocytes of both WT and *Atg5^K^*^O^ mice. (d, f) EM images of reticulocytes containing autolysosomes digesting mitochondria from both WT and *Atg5^K^*^O^ mice. (g, i) A slight increase in mitophagy was observed in *Atg5^KO^* reticulocytes and erythrocytes. Furthermore, the number of persisting mitochondria in reticulocytes and erythrocytes of *Atg5^KO^* mice was comparable to those of WT mice (h, j). The number of mitophagy events was calculated as the sum of autophagosomes and autolysosomes containing mitochondria. Error bars represent SD (*P* < 0.05; *n* = 55 cells).

During the fetal period, the liver functions as a hematopoietic organ. In mice, hematopoiesis is actively observed from embryonic day (E) 12.5. In particular, from E14.5 to E15.5, erythroblasts proliferate actively and typical heterochromatin appears on the nuclear membrane. Subsequently, the nuclei move toward the PM (Fig. 8a), and denucleation occurs ­(Fig. 8b). After denucleation, mitochondria are engulfed by isolation membranes, which fuse and become autophagosomes (Fig. 8c and e). The autophagosomes then fuse with lysosomes and degrade the mitochondria to become reticulocytes (Fig. 8d and f) in both WT and *Atg5^KO^* mice. Analysis of mitophagy demonstrated that mitochondria are induced and disappear from reticulocytes, albeit slightly later than in the WT mice (Fig. 8g–j) [11,44]. This intricate process demonstrates the integral role of GOMED in orchestrating mitochondrial clearance during erythrocyte maturation.

## *Wipi3* conditional knockout (cKO) mice show neurological defects owing to Fe deposition and Purkinje cell death

To further compare canonical autophagy and GOMED, we performed ultrastructural analysis in the cerebellum of *Atg7^cKO^* mice and *Wipi3^cKO^* mice [42]. Both strains demonstrated similar neurological defects [45]. However, we found substantial morphological differences between the two strains.

**Fig. 9.**
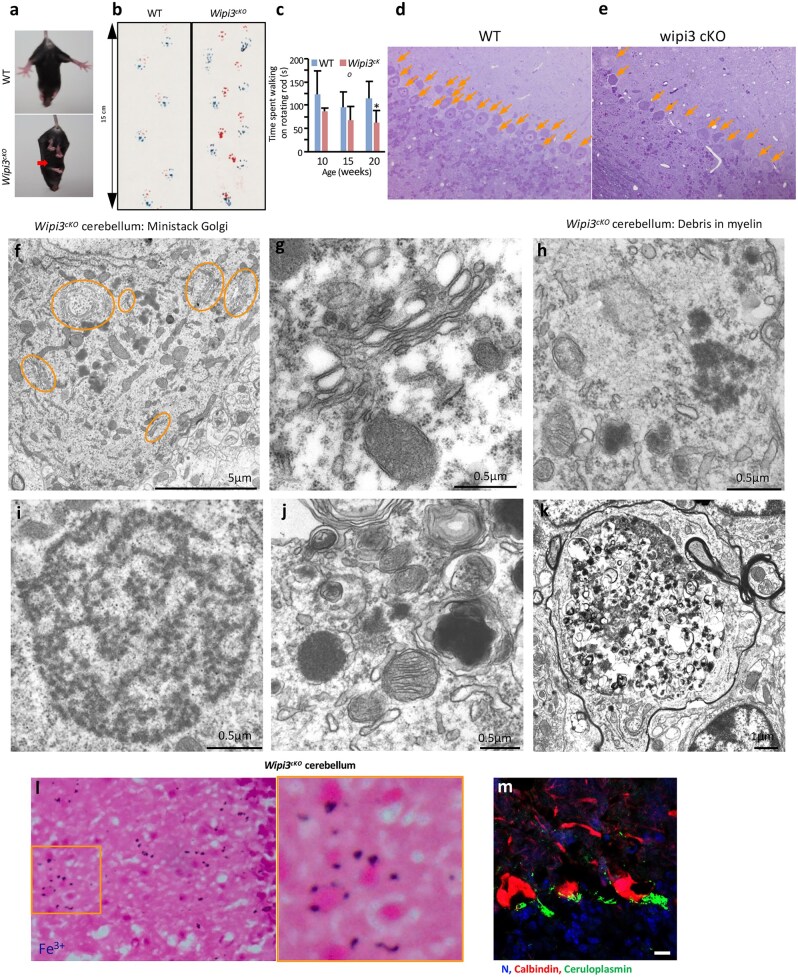
Neurological defects in neuron-specific *Wipi3^cKO^* mice. (a–c) Abnormal motor performance in *Wipi3^cKO^* mice at 10 weeks. The limb-clasping reflex was observed (a). The footprint assay indicated a motor deficit (b). In (c), the time the indicated mice remained on a rotating rod was measured. Data are shown as the mean ± SD (*n* = 3). (d, e) Semithin sections of the cerebellum of the indicated mice were stained with toluidine blue. Arrows indicate Purkinje cells, which were decreased in *Wipi3^cKO^* mice compared with WT mice. (f, g) Morphology of the Golgi apparatus in Purkinje cells of *Wipi3^cKO^* mice. (f) Orange circles indicate ministacked Golgi. (g) Ministacked Golgi showing swelling and abnormal structures. (h–k) The accumulation of dense fibrils was observed in Purkinje cells from *Wipi3^cKO^* mice, which are the most prominent abnormal structures observed in these mice. Bars = 0.5 μm. Dense fibrils imperfectly enclosed by autophagic membranes were also observed (j, k). (l, m) Accumulation of iron and ceruloplasmin in neuron-specific *Wipi3^cKO^* mice. (l) Cryosections of the cerebellum of the indicated mice were stained with Prussian blue. (m) Sections were immunostained with anti-ceruloplasmin (green) and anti-calbindin (red) antibodies. Blue puncta indicate iron deposition in (l). (a)–(c) were modified from Fig. 7b–d, respectively of ref. [42]. (i) and (m) were modified from Fig. 8a and b, respectively. (f)–(k) were obtained from the same experiments as those shown in Fig. 7i and k–m.

In the Purkinje cells of *Wipi3^cKO^* mice, we observed the accumulation of small, fragmented and swollen rod-shaped Golgi membranes (Fig 9f and g) [42], which was reminiscent of the morphology observed in etoposide-treated *Wipi3^KO^* MEFs. Conversely, the Golgi apparatus of *Atg7^cKO^* Purkinje cells was intact and extensive, while smooth ER membranes were highly enriched, which is a feature not observed in *Wipi3^cKO^* Purkinje cells [42].

## Loss of autophagy leads to the abnormal accumulation of substances in the cytoplasm

Massive inclusion bodies have been reported to be observed in the cerebella of *Atg7^cKO^* mice [42,45]. However, almost no such inclusion bodies were observed in the cerebella of *Wipi3^cKO^* mice [42]. In contrast to the inclusion bodies, we observed many deep black-stained fibrils enveloped by imperfect autophagic membranes in the *Wipi3^cKO^* mouse cerebellum, which is a feature not observed in *Atg7^cKO^* mice (Fig. 9h–k) [42].

Further analysis of the accumulated substances by Prussian blue staining revealed iron deposition primarily in the Purkinje and granular cells of *Wipi3^cKO^* mice (Fig. 9l), whereas *Atg7^cKO^* mice did not demonstrate such iron deposition [42]. Iron deposition is considered to be a possible trigger of several neurodegenerative diseases, such as neurodegeneration with brain accumulation, and abnormal iron deposition is usually associated with the abnormal accumulation of iron-binding proteins [46–48].

We hence analyzed the expression of iron-binding proteins in these mice by immunostaining and found that ceruloplasmin expression was increased in the brain, particularly in Purkinje cells and granule cells of *Wipi3^cKO^* mice (Fig. 9m) [42].

## Conclusion

Over 15 years of research, we have employed EM and other methods to identify a novel macroautophagy system that utilizes the Golgi apparatus, which we have named GOMED. The morphology of GOMED is characterized by a sequestration membrane that is derived from the Golgi membrane, which envelops organelles, such as mitochondria and secretory granules in the cytoplasm as it forms an autophagosome, and these organelles are degraded upon fusion of the autophagosome with a lysosome. This is in contrast to canonical autophagy, in which the isolation membrane originates mainly from the ER membrane.

The discovery of GOMED provides new insights into cellular degradation systems. The distinct origins of sequestration membranes between canonical autophagy and GOMED may be attributed to differences in their *in vivo* functions and the substrates that they degrade. As expected, GOMED plays a crucial role in the elimination of mitochondria and secretory proteins.

We have identified *Wipi3* as an essential gene for GOMED. By observing neuron-specific *Wipi3^cKO^* mice, we demonstrated that the accumulation of iron and ceruloplasmin leads to neurodegeneration. Furthermore, we have clarified that the overexpression of Dram1, a Golgi-localized molecule that activates GOMED [49], can rescue the GOMED defect in *Wipi3^KO^* MEFs and ameliorate the neurological phenotypes of *Wipi3^cKO^* mice [42]. This suggests that the neurological defects in *Wipi3*^*cKO*^ mice are owing to GOMED dysfunction and that Dram1 acts downstream of Wipi3. These findings suggest that Wipi3 is an essential molecule for GOMED and protects neurons through mechanisms distinct from canonical autophagy.

However, we have not yet identified the precise molecular mechanisms of GOMED. Our group’s research demonstrates distinct regulatory mechanisms between GOMED and canonical autophagy through differential phosphorylation of Ulk1 during DNA damage responses [23]. While both pathways require PPM1D-mediated dephosphorylation of Ulk1 at Ser637 (initiated by p53 activation), GOMED specifically involves RIPK3-dependent phosphorylation of Ulk1 at Ser746 in the cytosol [50,51]. This phosphorylation triggers Ulk1 translocation to Golgi membranes, where it recruits Wipi3 through PI3P-dependent mechanisms. Wipi3 then mediates the formation of curved isolation membranes from ministacked Golgi membranes [42].

Lastly, we found that GOMED utilizes the Golgi membrane for organelle clearance, particularly in the degradation of secretory granules and mitochondria in erythrocytes [11,12,44]. This suggests that GOMED may function more prominently in the secretion of hormones and the differentiation of other blood cells during the differentiation period.

Several studies on alternative types of autophagy have been published to date, some of which suggest that these pathways are involved in specific diseases and play important physiological roles [16,19,28–31]. As autophagy is a redundant process, it has been argued that alternative autophagy only occurs when canonical autophagy is deficient, i.e. it acts as a compensatory mechanism for canonical autophagy. This argument was based on the fact that no specific markers or regulatory molecules of GOMED had been identified. However, we identified the GOMED-specific regulatory molecule Wipi3 and demonstrated that the phenotypes of *Wipi3^KO^* mice and those of *Atg7^KO^* mice are clearly distinct. Furthermore, differences in the accumulated cellular components in each of the KO mice suggest that the substrates targeted by canonical autophagy and GOMED are distinct from each other. Therefore, these results have led to the growing recognition of GOMED as an alternative autophagy pathway with distinct roles from canonical autophagy. These insights indicate the importance of considering alternative autophagy pathways, such as GOMED, in future research, to better understand the complexity and diversity of autophagic processes.
